# Metagenomics next-generation sequencing of plasma combined with blood cells for improving the prognosis of early infection in patients with hematologic disorders: a real-world cohort study in northern China

**DOI:** 10.3389/fmolb.2026.1662559

**Published:** 2026-06-10

**Authors:** Cuicui Lyu, Qianyi Zhou, Xia Xiao, Xue Bai, Yedi Pu, Haibo Zhu, Mingfeng Zhao, Juanxia Meng, Hairong Lyu

**Affiliations:** 1 Department of Hematology, Tianjin First Central Hospital, School of Medicine, Nankai University, Tianjin, China; 2 First Center Clinical College, Tianjin Medical University, Tianjin, China

**Keywords:** blood cells, hematologic disorder, infection, next-generation sequencing, plasma

## Abstract

**Introduction:**

Infection is a leading cause of death in hematologic disorder patients. While plasma metagenomic next-generation sequencing (mNGS) is widely used, no studies have explored the clinical value of whole blood mNGS, combining plasma and blood cells, in these patients.

**Methods:**

We retrospectively analyzed the results of whole blood mNGS testing from 231 blood samples of hematological disorders patients with suspected infections. The diagnostic performance of whole blood mNGS and its clinical impacts on treatment were assessed based on the final clinical diagnosis.

**Results:**

mNGS testing in both plasma and whole blood showed significantly higher pathogen detection rates than blood culture (72.29%, 77.06% vs. 21.65%, P < 0.001). The total concordance rate of whole blood mNGS was also significantly higher than that of blood culture, conventional microbial testing, and plasma mNGS when compared to the final clinical diagnosis. Of the 101 pathogens detected by whole blood mNGS, 13 were missed by plasma mNGS. As a result, whole blood mNGS demonstrated a broad pathogen detection capability, especially in patients with non-hematologic malignancies or hematopoietic stem cell transplantation. Regarding treatment, whole blood mNGS had a positive impact on 72.73% of all patients, and 75.15% patients with pulmonary infections. It helped rule out infection in a timely manner, reduce or stop unnecessary antibiotic use, and enabled 77.88% of infected patients to benefit from whole blood mNGS sequencing.

**Discussion:**

Whole blood mNGS assays, combining plasma and blood cells, significantly improved pathogen detection rates and optimized antibiotic therapy in patients with hematological diseases and pulmonary infections or bloodstream infection. This approach facilitates the early management of patients with hematologic disorders who are at risk of infection.

## 1 Introduction

According to the most recent Global Burden of Disease Study 2021 (GBD 2021) and Global Cancer Observatory (GLOBOCAN) 2022 data, hematological malignancies continue to represent a significant global health burden. In 2022, hematological malignancies accounted for 6.6% of total global cancer cases (Bray et al., 2024; Pan et al., 2025). These malignancies contributed to 7.2% of total cancer-related deaths worldwide (Pan et al., 2025). From 1990 to 2021, global hematological malignancy cases showed a continuously increasing trend, reaching approximately 1.34 million incident cases in 2019 (Zhang N. et al., 2023). The primary causes for the increase include rapidly expanding elderly populations, enhanced diagnostic capabilities and increased global population base (Zhang X. et al., 2023; An et al., 2023). Infections are common in patients with hematologic disorders, particularly hematologic malignancies, due to increased susceptibility from abnormal hematopoietic function (Moreno-Sanchez and Gomez-Gomez et al., 2022). In recent years, advancements in treatments for hematologic diseases (such as targeted therapy and immunotherapy) have improved both survival rates and the duration of survival (Wang, 2020). However, these treatments may also increase the risk of infections. Postoperative fever is a common symptom in patients undergoing treatment for hematologic disorders (Corkum et al., 2018). However, not all fevers indicate an infection. In these patients, the symptoms of infection are often subtle in the early stages. Failure to identify infection promptly and accurately can lead to high mortality (Xu et al., 2023). Timely pathogen detection in the early stages is crucial for optimizing antibiotic treatment (Schmidt-Hieber et al., 2019). However, diagnosing early infection in hematologic disorder patients is challenging due to the low sensitivity of non-specific clinical signs and conventional microbiological tests (CMT) (Feng et al., 2024; Zhang N. et al., 2023).

Culture, an important tool for the clinical diagnosis of infection, has a long turnaround time and is affected by prior antibiotic exposure, which hinders early infection diagnosis. Polymerase Chain Reaction (PCR) and serological antigen tests continue to be the gold standard for viral testing as part of CMT (Xu et al., 2023). Recent studies have shown that reactivation of latent viruses is common in hematologic disorders, leading to a significant increase in the detection rate of these viruses (de Melo Silva et al., 2020; Lu et al., 2021). However, the clinical significance of these positive results for latent viruses may complicate treatment decisions. Recent advances in next-generation sequencing (NGS) have paved the way for research on microbial community composition. Numerous studies have demonstrated the clinical utility of NGS, leading to more tailored treatments and potentially better outcomes for patients (Carbonell et al., 2019; Kalbitz et al., 2024). Despite these advancements, mNGS has several limitations. First, interference from the large amount of host DNA (>90%–99%) significantly impacts the detection of minuscule Cell-free DNA (cfDNA). Second, the short half-life of cfDNA can reduce the sensitivity of cfDNA mNGS (Chen et al., 2023). Finally, cfDNA is fragmented and lacks a complete genome sequence, which may lead to overlooked information about drug resistance (Wang D. et al., 2024). In previous studies, the combination of cfDNA and cellular DNA for different body fluid samples increased the sensitivity of mNGS (Chen et al., 2023; Wu et al., 2023). However, the advantages and disadvantages of this combined approach compared with plasma cfDNA detection in early infection in patients with hematologic disorders remain undetermined.

Although plasma cfDNA mNGS has been used to detect a variety of hematologic disorders, both plasma cfDNA and blood cells mNGS have also been applied to detect other conditions, such as sepsis (Wu et al., 2023; Zhu et al., 2025). However, previous studies have primarily focused on pathogen identification, with few integrating testing methods with treatment. This study was designed to systematically compare the diagnostic performance of plasma mNGS and whole blood mNGS (plasma combined with blood cells) against the final clinical diagnosis in patients with hematologic disorders suspected of infection. Additionally, it sought to evaluate the impact of whole blood mNGS on therapeutic decision-making in both infected and non-infected patients, thereby providing a comprehensive assessment of its clinical application value in patients with hematologic disorders.

## 2 Materials and methods

### 2.1 Patient enrollment and study design

We conducted a retrospective analysis of patients who underwent whole blood mNGS testing for suspected infection from June 2022 to March 2024 in the Department of Hematology, Tianjin First Central Hospital. This study was approved by the Ethics Review Committee of Tianjin First Central Hospital. Given the retrospective nature of the study, the ethics committee waived the requirement for informed written consent, and all patient information was anonymized. The inclusion criteria are as follows: 1) Diagnosis of hematological diseases, mainly including leukemia, lymphoma, multiple myeloma, aplastic anemia, *etc.*, with treatment involving medications, immunosuppressants, or glucocorticoids; 2) Patients with a high suspicion of clinical infection; 3) Blood samples were collected for both mNGS and blood culture testing simultaneously (Wang X. et al., 2024); (4) There were no age restrictions; all patients who met the other inclusion criteria were enrolled. Exclusion criteria: 1) Patients with non-hematologic disorders; 2) mNGS and blood culture were not performed simultaneously; 3) Missing or incomplete case information.

The final clinical diagnosis of infection was evaluated by two supervising clinicians based on the composite reference standard: combined results of all microbiological tests, radiological testing results, and a summary of the patient clinical examining results and symptoms. When the assessments differed, a third supervising expert was invited to make the final decision. The clinical diagnostic for bloodstream infections (BSIs) was primarily based on “Chinese Expert Consensus on Clinical Protocols for Blood Culture in Bloodstream Infection Diagnosis” (Wang and Wang, 2022). Lower Respiratory Tract Infections (LRTIs) were diagnosed in accordance with the Guidelines for the Management of Adult Lower Respiratory Tract Infections (Woodhead et al., 2011).

In this study, immunocompromised status was defined based on the following criteria applicable to patients with hematological diseases: 1) Hematologic malignancies (including leukemia, lymphoma, and multiple myeloma); 2) Neutropenia (including aplastic anemia) or currently receiving chemotherapy; 3) Long-term use of corticosteroids (such as patients with idiopathic thrombocytopenic purpura (ITP)). Criteria related to solid organ transplantation, solid tumor chemotherapy, antirheumatic drugs, or biological immunomodulators were not applicable to our study cohort and were therefore excluded from the assessment (Ramirez et al., 2020).

### 2.2 Specimen collection and conventional microbiological tests

Clinical specimens, including blood, sputum, urine, feces, secretions, alveolar lavage, cerebrospinal fluid, pus, throat swabs, anal swabs, skin swabs, nasal swabs, and ear swabs, were collected as soon as infection was suspected and promptly sent to the clinical laboratory department for CMT. For blood samples, both blood culture (BC) and mNGS were performed to detect pathogenic microorganisms. For other specimen types, CMT was selectively conducted based on the patient’s clinical presentation. CMT included: culture, 1.3-β-D-glucan (G test), galactomannan (GM test), polymerase chain reaction (PCR) for cytomegalovirus (CMV), Epstein-Barr virus (EBV), Severe Acute Respiratory Syndrome Coronavirus 2 (SARS-CoV-2), human polyomavirus 1 (BKV), and hepatitis B virus (HBV), as well as serological antibody testing for CMV, EBV, influenza A virus, influenza B virus, *Mycoplasma pneumoniae* (MP), SARS-CoV-2, and *Aspergillus* spp.

### 2.3 mNGS sequencing and analysis

The turnaround time (from sample receipt to report generation) for mNGS testing ranged between 15 and 20 h, with a median of 17.5 h. The workflow for mNGS testing of whole blood samples is as previously described (Chen et al., 2023). DNA extraction was performed using the magnetic bead-based method. Whole blood (5 mL) was centrifuged at 1900 *g* at 4 °C for 10 min to separate the plasma and blood cells. cfDNA was then extracted from the plasma using the PathoXtract® mcfDNA Pathogen Extraction Kit (WYXM03010S, Willingmed Corp, Beijing, China). Pathogenic genomic DNA was extracted from the blood cells using the PathoXtract® HemoCyte Whole Blood Pathogen Enrichment Extraction Kit (WYXM03210S, Willingmed Corp, Beijing, China). The nucleic acid extraction procedure of this kit initiates with the selective lysis of blood cells using saponin (at a final concentration of 2.2%), releasing intracellular nucleic acids into the solution. Subsequently, centrifugation is performed to remove the lysed cellular background, thereby enriching pathogens in the pellet. The pellet is then resuspended in a bacterial lysis buffer and subjected to bead beating to efficiently disrupt the cell walls of difficult-to-lyse microorganisms, ultimately releasing pathogen-derived nucleic acids for downstream analysis.

The cfDNA and blood cell genomic DNA samples were then processed using KAPA Hyper Prep Kits (KK8504, KAPA, Kapa Biosystems, Wilmington, MA, United States) and Illumina® DNA Prep (M) Tagmentation (20018705, Illumina) according to the manufacturer’s instructions to construct the library. This was followed by 75 bp single-end read sequencing on the NextSeq™ 550Dx (Illumina), with no fewer than 20 million reads per sample.

Trimmomatic v0.40 was used to eliminate low quality reads, contaminated joints, duplicate readings, and short reads (Bolger et al., 2014). High-quality reads were aligned to the human reference genome GRCh2 (hg19) using Bowtie2 v2.3.5 software to remove human sequences (Langmead and Salzberg, 2012). The remaining clean non-host reads were then used for pathogen classification and identification using Kraken2 v2.1.0 against a comprehensive pathogen database derived from NCBI GenBank, RefSeq, and nt collections (Wood et al., 2019). Pathogen positivity is determined by the value per ten million readings (RPTM). Following previously validated thresholds for clinical mNGS interpretation (Wang D. et al., 2024; Wu et al., 2023; Ma et al., 2025), viruses were considered positive when RPTM ≥3; bacteria and fungi required RPTM ≥8 for positivity determination. For specific pathogens with high clinical significance including *cryptococcus* and *mycobacteria,* a lower threshold of RPTM ≥1 was applied due to their clinical importance and typically low abundance in clinical samples.

### 2.4 Clinical impact of mNGS on treatment management

Combining laboratory and clinical data, mNGS results were classified as definite, probable, possible, and unlikely, based on a combination of microbiological and clinical criteria outlined in the Karius test (Chen et al., 2023; Blauwkamp et al., 2019). In our study, we classified definite, probable and possible microorganisms as clinically relevant microorganisms, while unlikely microorganisms were classified as colonization or contamination. In this study, the effect of whole blood mNGS on patient treatment was categorized into three levels: positive, negative, and no effect (Xu et al., 2023; Xu X. et al., 2024). Positive effects suggest that mNGS can help with antibiotic modulation [including initialization of the appropriate antibiotics treatment (M1), antibiotic escalation (M2), and antibiotic de-escalation (M3)] or demonstrate the effectiveness of empiric antimicrobial therapy (M4). No effect means that antibiotic therapy has not been clinically adjusted despite mNGS results were positive (M5) or negative (M7), or the patient was discharged or died before receiving mNGS results (M6). Negative effects indicate unnecessary treatment based on the results of mNGS (M8).

### 2.5 Statistics analysis

Statistical analysis was performed using SPSS 26.0 software (IBM, Armonk, NY) and GraphPad Prism 8.0 (GraphPad, La Jolla, CA). Continuous variables are represented by medians and ranges or mean ± standard deviation, while categorical variables are represented by counts and percentages. Depending on the data type and distribution, differences between groups were analyzed using the Student’s t-test, chi-square test, or Fisher’s exact test. A 2 × 2 contingency table was constructed to calculate sensitivity, specificity, positive predictive value (PPV) and negative predictive value (NPV). A *P* value <0.05 denoted statistical significance.

## 3 Results

### 3.1 Patient characteristics

**FIGURE 1:**
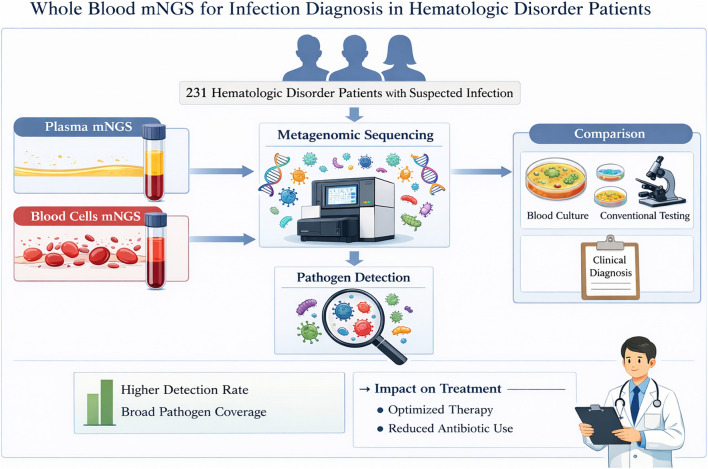
Workflow of whole blood mNGS.

Between June 2022 and March 2024, a total of 302 individuals who underwent whole blood mNGS testing were screened, of whom 231 met the inclusion criteria were included in the final analysis. The workflow of whole blood mNGS was showed in Figure 1. Table 1 summarizes the baseline characteristics and clinical test results of the patients. The median age was 40 years (range, 26–58), with 58.44% (135/231) being male. Hypertension and diabetes were present in 18.61% (43/231) and 17.32% (40/231) of the participants, respectively. The majority had hematologic malignancies, primarily acute myeloid leukemia (AML, 48.48%, 112/231), acute lymphoblastic leukemia (ALL, 18.18%, 42/231), and myelodysplastic syndromes (MDS, 16.02%, 37/231). Overall, 64.07% (148/231) underwent hematopoietic stem cell transplantation (HSCT), and 98.27% (227/231) were immunosuppressed. The in-hospital mortality rate was 19.05% (44/231), with a median length of hospital stay of 26 days (IQR 14–37.5) (Table 1).

**TABLE 1:** Clinical characteristics of 231 enrolled patients.

Demographics	Case (n = 231)	Infectious group (n = 208)	Non-infectious group (n = 23)	*P* value
Age (y)				
Median (range)	40 (26–58)	45 (32–60)	33 (18–44)	0.021*
Distribution, n (%)				
0–20	43 (18.61%)	35 (16.83%)	8 (34.78%)	0.048*
21–40	64 (27.71%)	56 (26.92%)	8 (34.78%)	0.464
41–60	75 (32.47%)	71 (34.13%)	4 (17.39%)	0.157
>60	49 (21.21%)	46 (22.12%)	3 (13.04%)	0.426
Male sex, n (%)	135 (58.44%)	122 (58.65%)	13 (56.52%)	0.828
Fever, n (%)	196 (84.85%)	174 (83.65%)	22 (95.65%)	0.216
Hematologic diseases				
Acute myeloid leukemia (AML)	112 (48.48%)	102 (49.04%)	10 (43.48%)	0.665
Acute lymphoblastic leukemia (ALL)	42 (18.18%)	38 (18.27%)	4 (17.39%)	>0.999
Acute mixed lineage leukemia (MAL)	4 (1.73%)	4 (1.92%)	0 (0.00%)	>0.999
Myelodysplastic syndromes (MDS)	37 (16.02%)	31 (14.90%)	6 (26.09%)	0.225
Aplastic anemia (AA)	11 (4.76%)	9 (4.33%)	2 (8.70%)	0.301
Chronic lymphocytic leukemia (CLL)	2 (0.87%)	2 (0.96%)	0 (0.00%)	>0.999
Malignant lymphoma	11 (4.76%)	10 (4.81%)	1 (4.35%)	>0.999
Multiple myeloma (MM)	4 (1.73%)	4 (1.92%)	0 (0.00%)	>0.999
Others (ITP,etc.)	6 (2.60%)	6 (2.88%)	0 (0.00%)	>0.999
Transplantation	148 (64.07%)	129 (62.02%)	19 (82.61%)	0.066
Immunosuppression	227 (98.27%)	204 (98.08%)	23 (100.00%)	>0.999
Discharge outcome				
Survival	187 (80.95%)	164 (78.85%)	23 (100.00%)	0.010**
Death	44 (19.05%)	44 (21.15%)	0 (0.00%)	0.010**

y, year; LOHS, length of hospital stay, “Others” under hematologic disorders category including one case of idiopathic thrombocytopenic purpura (ITP), three cases of anemia, and two cases of hemophagocytic syndrome.*P < 0.05. ** *P* < 0.01

Based on the final clinical assessment, 90.04% (208/231) of the patients were diagnosed with infection and were classified into the infection group, while the remaining 9.96% (23/231) were classified as the non-infected group. Patients in the infection group were significantly older than non-infected patients (45 vs. 33, *P* = 0.021) and had significantly higher mortality rates (21.15% vs. 0.00%, *P* = 0.010) (Table 1).

### 3.2 Detection performance of whole blood mNGS and CMT

**FIGURE 2:**
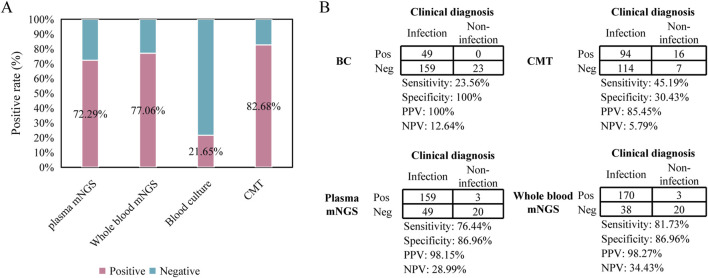
Diagnostic performance of plasma mNGS, whole blood mNGS, BC, and CMT in patients with hematologic disorders. **(A)** Overall positive rates of plasma mNGS, whole blood mNGS, BC and CMT (includes BC) across all enrolled patients. **(B)** Contingency tables and diagnostic metrics for BC, CMT, plasma mNGS, and whole blood mNGS in distinguishing infection from non-infection patients with hematologic disorders. Sensitivity, specificity, positive predictive value (PPV), and negative predictive value (NPV) are shown for each method. Note that CMT incorporates BC results alongside other conventional microbiological tests. Pos, positive; Neg, negative.

Among the 231 enrolled patients, whole blood mNGS yielded a positive rate of 77.06%, which was significantly higher than that of blood culture (BC) (21.65%, *P* < 0.001), but no significantly different from plasma mNGS (72.29%, *P* = 0.285) or CMT (82.68%, *P* = 0.132) (Figure 2A). Using the final clinical diagnosis as the reference standard, whole blood mNGS achieved the highest sensitivity, 81.73%, followed by plasma mNGS (76.44%), CMT (45.19%) and BC (23.56%). BC had the highest specificity, 100%, followed by whole blood mNGS and plasma mNGS (both 86.96%). Whole blood mNGS also demonstrated a higher positive predictive value (PPV, 98.27%) and negative predictive value (NPV, 34.43%) compared with the other methods (Figure 2B).

**FIGURE 3:**
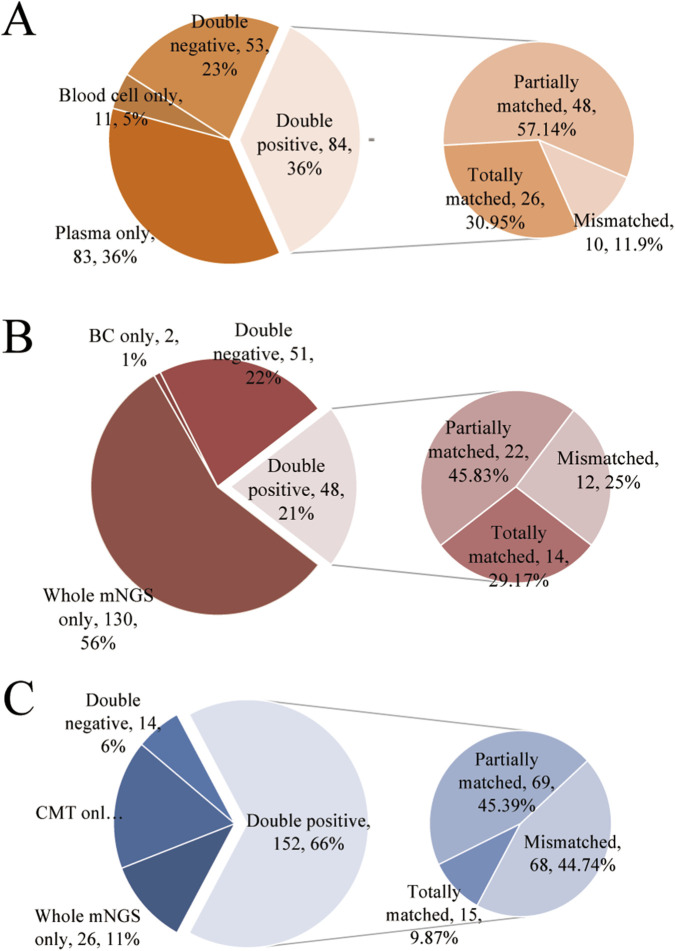
Inter-method agreement in pathogen detection. **(A)** Concordance between plasma and blood cell mNGS. **(B)** Agreement between whole blood mNGS and BC. **(C)** Agreement between whole blood mNGS and CMT.

Among whole blood mNGS-positive patients, 36% had concordant positive results from both the plasma and blood cells fractions, with an overall concordance rate of 88.10% (including both totally + partially matched) (Figure 3A). Compared to BC, 1% (2/231) of the samples tested positive by BC alone. Due to the low positive rate of BC, only 21% of the samples were co-positive for both whole blood mNGS and BC, with a pathogen-level agreement of 75% (Figure 3B). When whole blood mNGS results were combined with CMT, 94% of patients had at least one positive result, and 66% tested positive by both methods. Despite this high co-positivity rate, the pathogen-level agreement between whole blood mNGS and CMT was relatively low at 55.26% (Figure 3C).

### 3.3 Spectrum of clinically relevant pathogens

**FIGURE 4:**
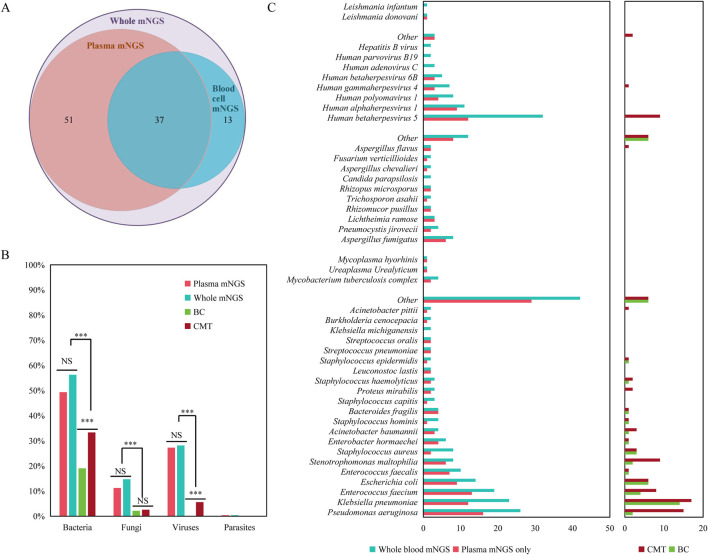
Characterization of pathogen detection across mNGS, BC and CMT. **(A)** Comparison of pathogen types identified by the plasma and blood cells mNGS assays. **(B)** Detection rates of plasma mNGS, whole blood mNGS, BC and CMT across pathogen categories. NS, No statistically significant difference. ***, *P* < 0.001. **(C)** Spectrum of pathogens detected by plasma mNGS, whole blood mNGS, BC and CMT. CMT, conventional microbiological testing (incorporating BC results).

A total of 107 clinically relevant microorganisms were identified across all patients by whole blood mNGS, BC and CMT (including BC), comprising 69 bacterial species (including 3 atypical pathogens), 24 fungal species, 12 viral species, and two parasitic species. Whole blood mNGS demonstrated superior detection capability, identifying 101 of the 107 total species (94.4%). When whole blood mNGS was partitioned into its plasma and cellular components, plasma mNGS detected 88 species (82.2%), while an additional 13 clinically relevant microorganisms (12.1%) were identified exclusively in the blood cell fraction (Figure 4A). Whole mNGS and Plasma mNGS demonstrated superior diagnostic performance compared to CMT and BC for bacteria, fungi, and viruses (*P* < 0.001). While Whole mNGS consistently yielded the highest detection rates, its performance did not differ significantly from Plasma mNGS (*P* > 0.05). In contrast, traditional methods (CMT and BC) showed significantly lower sensitivity, particularly for viruses (5.63% and 0.00%, respectively). No significant differences were found in fungal detection between BC and CMT (*P* > 0.05), nor in parasite detection across all four methods (*P* > 0.05), where rates remained below 1% (Figure 4B).

The pathogen spectrum varied by detection method. Whole blood mNGS outperformed plasma mNGS in detecting the majority of pathogens, whereas CMT and BC showed relatively lower overall pathogen detection, with reasonable sensitivity limited primarily to bacterial organisms. Among infected hematologic disorder patients, the most common identified microorganisms were *Pseudomonas aeruginosa*, *Klebsiella pneumoniae*, and *human* herpesvirus *type 5* (CMV). In addition, the most common bacteria detected by plasma mNGS were *P. aeruginosa* and *Enterococcus faecium*, fungi were *Aspergillus fumigatus* and *Lichtheimia ramose*, and viruses including CMV and *human herpes simplex virus* 1 (HSV-1) (Figure 4C; Supplementary Table 1).

The 13 species identified exclusively by blood cells including 10 bacteria and 3 fungi (Figure 4C). Notable bacterial detections included *Corynebacterium pseudodiphtheriticum*, *Haemophilus influenzae*, *Aeromonas caviae*, *Roseomonas gilardii*, *Nocardiopsis dassonvillei*, *Burkholderia gladioli*, *Mycobacteroides immunogenum*, *Nocardia farcinica*, *Pseudomonas lundensis* and *Serratia marcescens.* Fungal species unique to blood cell mNGS included *Candida intermedia*, *Aspergillus ruber* and *Fusarium coffeatum.*

### 3.4 Detection performance of whole blood mNGS in patients with different clinical conditions

To further evaluate the diagnostic performance of whole blood mNGS across different clinical subgroups, patients were stratified by fever status, hematologic malignancy type, immune status, and HSCT history. Using the final clinical diagnosis as the reference standard, whole blood mNGS achieved a sensitivity above 72% and specificity above 84% across all subgroups, with the highest sensitivity observed in patients with non-hematologic malignancies (93.33%) and those who had undergone HSCT (87.60%). Positive predictive value (PPV) exceeded 97% in all subgroups, while negative predictive value (NPV) remained below 67% (Table 2).

**TABLE 2:** Comparison of diagnostic performance of whole blood mNGS across different patient populations.

Groups	Sensitivity (%)	Specificity (%)	PPV (%)	NPV (%)
Fever	82.18%	86.36%	97.95%	38.00%
Non-fever	79.41%	100.00%	100.00%	12.50%
Hematological malignancies	80.83%	85.71%	98.11%	32.73%
Non-hematological malignancies	93.33%	100.00%	100.00%	66.67%
Transplantation	87.60%	84.21%	97.41%	50.00%
Non-transplantation	72.15%	100.00%	100.00%	15.38%
Immunosuppression	81.37%	86.96%	98.22%	34.48%

HSCT, hematopoietic stem cell transplantation; PPV, positive predictive value; NPV, negative predictive value.

### 3.5 Clinical impact of whole blood mNGS on treatment

A comparative analysis was performed to evaluate the influence of various diagnostic methods on clinical treatment decisions. Whole blood mNGS demonstrated the highest proportion of positive influence (72.73%), surpassing plasma mNGS (68.40%), CMT (incorporating BC, 37.66%), and BC (18.61%). Compared with plasma mNGS, the added benefit of whole blood included confirmed empirical therapy (n = 5), initiation of appropriate antibiotics therapy (n = 2), antibiotic de-escalation (n = 2), and escalation (n = 1). For all methods, the most common reason for no impact was the absence of antibiotic adjustment when results were negative. Regrettably, mNGS results could also lead to a negative impact (0.87%) (Table 3).

**TABLE 3:** Impact of whole blood mNGS on the treatment of patients with suspected infections.

Impact	Grade	Description	Plasma mNGS	Whole blood mNGS	BC	CMT
Positive	M1	Initialization of the appropriate antibiotics treatment	45 (19.48%)	47 (20.35%)	6 (2.60%)	23 (9.96%)
	M2	Antibiotic escalation	46 (19.91%)	47 (20.35%)	10 (4.33%)	20 (8.66%)
	M3	Antibiotic de-escalation	17 (7.36%)	19 (8.23%)	2 (0.87%)	3 (1.30%)
	M4	Confirmed empirical treatment	50 (21.65%)	55 (23.81%)	25 (10.82%)	41 (17.75%)
No effect	M5	No adjustment in treatment while the result was positive	16 (6.93%)	16 (6.93%)	2 (0.87%)	3 (1.30%)
	M6	The patient was discharged or died	5 (2.16%)	5 (2.16%)	4 (1.73%)	4 (1.73%)
	M7	No adjustment in treatment while the result was negative	50 (21.65%)	40 (17.32%)	182 (78.79%)	137 (59.31%)
Negative	M8	NGS led to unnecessary treatment	2 (0.87%)	2 (0.87%)	0 (0%)	0 (%)

### 3.6 Clinical value of whole blood mNGS for BSI and pulmonary infection patients

**FIGURE 5:**
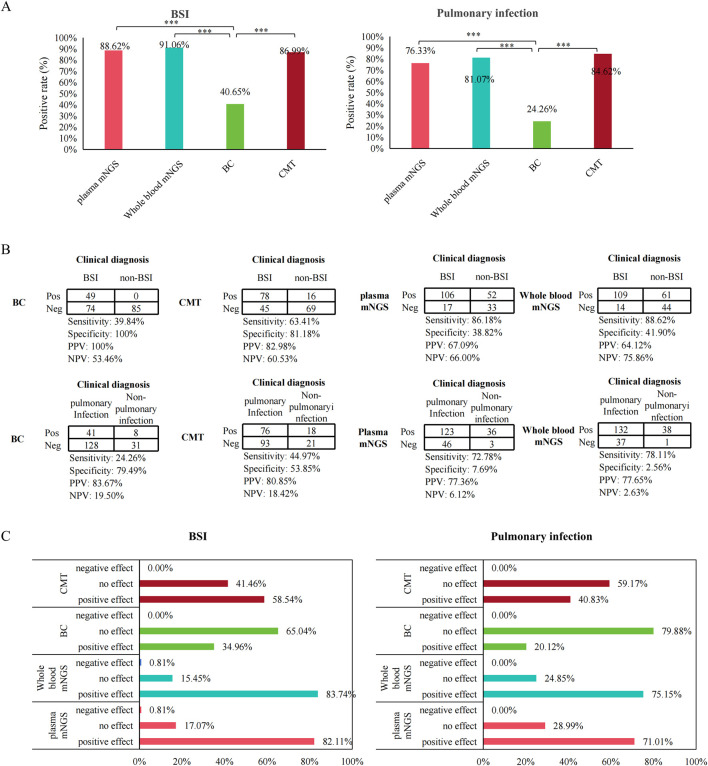
Clinical utility of whole blood mNGS compared with BC and CMT in patients with BSI and pulmonary infection. **(A)** Positive rates of different methods in BSI and pulmonary infection subgroups. NS, No statistically significant difference. ***, *P* < 0.001. **(B)** Contingency tables evaluating the diagnostic performance of each method for BSI and pulmonary infection. **(C)** Impact of each diagnostic method on clinical treatment decisions.

To further assess the clinical value of whole-blood mNGS in specific infection types, patients diagnosed with BSI and pulmonary infections were analyzed separately. For BSI patients, whole blood mNGS achieved the highest positive rate (91.06%), followed by plasma mNGS (88.62%), CMT (86.99%), and BC (40.65%). For patients with pulmonary infections, CMT had the highest positive rate (84.62%), followed by whole-blood mNGS (81.07%), plasma mNGS (76.33%) and BC (24.26%). In both infection types, the positive rates of plasma mNGS, whole blood mNGS, and CMT were each significantly higher than that of BC (all *P* < 0.001) (Figure 5A). Regarding diagnostic performance analysis, for BSI patients, the diagnostic sensitivity of whole-blood mNGS (88.62%) was higher than that of plasma mNGS (86.18%), CMT (63.41%), and BC (39.84%), with a consistent pattern observed in pulmonary infection patients (Figure 5B). Concerning clinical impact, for BSI patients, whole-blood mNGS had the highest proportion of positive impact on medication guidance at 83.74%, followed by plasma mNGS at 82.11%, CMT at 58.54%, and BC at 34.96%. For pulmonary infection patients, the positive impact proportions of whole-blood (75.15%) and plasma mNGS (71.01%) were also higher than those of CMT and BC (Figure 5C).

### 3.7 Enhancing mNGS performance in whole blood through additional blood cells detection

Two patients with special fungal infections diagnosed by blood cell mNGS were excluded from the cohort analysis due to missing synchronous blood cultures. These cases are presented separately to illustrate the clinical utility of whole blood mNGS—specifically the value of pathogen detection in the blood cell fraction—in diagnosing invasive fungal infections that were missed by conventional methods.

The key clinical features, mNGS findings, treatment adjustments, and outcomes for both patients are summarized in Table 4. In both cases, CMT and initial empirical therapy failed to identify or adequately cover the causative fungal pathogens. Whole blood mNGS, by simultaneously analyzing both plasma and blood cell fractions, detected clinically relevant fungi (*Rhizomucor pusillus* and *A. ruber*, respectively) that would otherwise have been missed. This enabled timely and targeted antifungal treatment adjustments, leading to clinical improvement in both patients. These findings highlight the added diagnostic value of including the blood cell fraction in whole blood mNGS workflows, particularly for immunocompromised patients at high risk of invasive fungal infections. The CT images before and after target treatment were showed in Supplementary Figure 1.

**TABLE 4:** Clinical summary of two patients with fungal infections diagnosed by blood cell mNGS.

Index	Patient 1	Patient 2
Underlying disease	Acute lymphoblastic leukemia (ALL)	Adult T-cell leukemia
Procedure	HSCT (5 months prior to admission)	HSCT (symptoms onset on day 7 post-HSCT)
Initial symptoms	High fever, thrombocytopenia, gum pain, oral blood blisters	High fever, chills
Initial CT findings	Inflammation of the upper lobe of the right lung	Inflammation of the lower lobe of the right lung
Conventional microbiological results	Throat swab: *Pseudomonas aeruginosa* (+)	Throat swab: *Pseudomonas aeruginosa* (+); anal swab: *Proteus mirabilis* (+)
Initial empirical therapy	Cefoperazone + polymyxin E + linezolid + caspofungin	Ceftazidime-avibactam sodium
Response to initial therapy	No improvement	No improvement; persistent high fever
mNGS – plasma fraction	*Pseudomonas aeruginosa*	*Pseudomonas aeruginosa*
mNGS – blood cell fraction	Rhizomucor pusillus	Aspergillus ruber
Treatment adjustment	Discontinued caspofungin; added posaconazole + amphotericin B (targeted anti-mucormycosis)	Added voriconazole (targeted antifungal therapy)
Clinical response	Body temperature normalized after 7 days; chest CT showed resolved lung inflammation	Body temperature decreased; inflammatory markers significantly improved
Follow-up imaging	Chest CT (Day 7): improvement in right upper lobe inflammation	Chest CT (Day 9): improvement in right lower lobe inflammation

## 4 Discussion

Infection remains a leading cause of mortality in patients with hematologic disorders, making accurate and timely pathogen identification essential for guiding treatment. In this study, whole blood mNGS demonstrated a substantially higher positive rate than BC (77.06% vs. 21.65%, *P* < 0.001), and showed superior sensitivity compared to BC, CMT, and plasma mNGS (Figure 2). Importantly, combinating the blood cells and plasma mNGS provided more comprehensive pathogen capture than plasma along, suggesting that the inclusion of supplemental blood cells contributes meaningfully to diagnostic yield.

Plasma samples primarily target cell-free DNA (cfDNA), which limits its ability to detect intracellular pathogens—such as Mycobacteroides and *Nocardia* species—due to the scarcity of microbial cfDNA in the extracellular compartment (Blauwkamp et al., 2019). Compounding this issue, host-derived DNA typically accounts for 90%–99% of total DNA in plasma, substantially diluting microbial signals and reducing overall detection sensitivity (Chen et al., 2023). Furthermore, cfDNA mNGS is prone to false-positive results, as transient microbial DNA can enter the bloodstream without representing true infection; one previous study reported that 60% of cfDNA mNGS-positive results were not confirmed by blood culture (Wang X. et al., 2024; Blauwkamp et al., 2019). To overcome these limitations, whole blood mNGS has been developed as an approach that incorporates both the plasma and cellular fractions. Our study found a 75% agreement in samples that were positive for both BC and whole blood mNGS (Figure 3), suggesting a lower false-positive rate than that previously reported for plasma cfDNA mNGS alone. Moreover, blood cells mNGS detected an additional 13 microorganisms associated with infection, including two obligate intracellular bacteria (*M. immunogenum*, *N. farcinica*) and three facultative intracellular bacteria (*C. pseudodiphtheriticum*, *H. influenzae* and *B. gladioli*) (Figure 4), thereby reducing the risk of false-negative results, however, that the blood cells was less effective in detecting viruses. This is consistent with previous studies, which show that cfDNA mNGS is better at detecting viruses than cellular DNA mNGS, while plasma mNGS alone is less effective than a combination of plasma and blood cells mNGS in detecting bacteria and fungi (Han et al., 2022). This may be due to the fact that some Gram-negative bacteria are easily lost during host clearance, while blood cells mNGS uses differential lysis to remove host nucleic acids, thereby enhancing detection sensitivity (Nelson et al., 2019; Marotz et al., 2018).

Compared with currently available molecular diagnostic approaches for bloodstream infection (BSI), whole-blood mNGS offers several distinct advantages. Nevertheless, multiple emerging technologies have also been developed to improve the rapid detection of pathogens in blood samples. Probe-capture targeted next-generation sequencing (tNGS) can identify more than 3,000 pathogens through the use of overlapping hybridization probes, enabling broader genomic coverage and more comprehensive target capture while reducing background or host DNA interference (Harris et al., 2025). Previous studies have reported a detection rate of 60.3% in patients with hematologic malignancies, exceeding that of conventional microbiological methods (Xu et al., 2024a). In patients with BSI, Probe-capture tNGS has demonstrated diagnostic sensitivity comparable to that of mNGS and superior to both blood culture and CMT (Wei et al., 2025). However, because the assay relies on predefined probe panels, it is unable to detect novel or highly divergent antimicrobial resistance determinants and pathogens that are not included in the panel, which may lead to false-negative results (Papamentzelopoulou et al., 2025). Droplet digital PCR (ddPCR) enables rapid absolute quantification of pathogens within 4–8 h while simultaneously detecting antimicrobial resistance gene. This method is characterized by low replication variability, a low detection limit, and high genus/species specificity. However, ddPCR typically targets only a limited spectrum of pathogens (several to dozens), and it does not provide information on antimicrobial susceptibility (Lin et al., 2023; Ziegler et al., 2019; Tedim et al., 2024; Wouters et al., 2020). T2 magnetic resonance (T2MR; T2 Biosystems, Lexington, MA, United States) is another technology capable of detecting pathogens directly from whole blood within 3–8 h. In this system, microbial DNA amplified by PCR hybridizes with probes coated on superparamagnetic nanoparticles, allowing the amplified products to be detected through changes in the magnetic resonance signal. Notably, the T2MR system detects intact microbial cells rather than free circulating DNA (Neely et al., 2013). Although studies have shown a relatively high concordance (approximately 80%) between pathogens detected by T2MR and those identified by blood culture in patients with BSI, the assay is restricted to a limited number of bacterial and fungal species (Nguyen et al., 2019; Bonura et al., 2024), making it insufficient as a standalone diagnostic tool for hematologic patients. PCR coupled with electrospray ionization mass spectrometry (PCR/ESI-MS), previously implemented on the commercial IRIDICA platform (Abbott Molecular, Des Plaines, IL, United States), represents another innovative approach combining PCR amplification with mass spectrometric analysis. This technology can directly detect more than 800 bacterial and *Candida* species associated with BSI from whole-blood samples and can shorten the turnaround time to approximately 6 h (Bacconi et al., 2014). Despite demonstrating better diagnostic performance than blood culture, the IRIDICA system is no longer commercially available because its production has been discontinued (Tkadlec et al., 2020; Sinha et al., 2025). In addition, microfluidic systems integrated with biosensor-based pathogen detection have emerged as promising tools for rapid infectious disease diagnostics. Microfluidic chips offer several advantages, including short detection times, minimal sample requirements, portability, operational simplicity, and the potential for multifunctional integration. However, many current microfluidic diagnostic platforms remain at the proof-of-concept or developmental stage due to high technical requirements and the lack of standardized operational procedures (Lehnert and Gijs, 2024; Asghar et al., 2019). In contrast to these targeted or platform-specific technologies, whole-blood mNGS provides hypothesis-free, broad-spectrum pathogen detection across bacteria, fungi, viruses, and parasites. Importantly, it can capture intracellular and cell-associated microorganisms that may be missed by plasma-based sequencing approaches. This advantage was illustrated in the present study, in which 13 additional pathogens were detected exclusively in the blood cell fraction. Each diagnostic method has its own strengths and limitations, and the optimal approach should be selected according to specific clinical scenarios. In China, ddPCR, tNGS, and mNGS are currently among the most widely used molecular pathogen detection methods. Clinical guidelines and expert consensus recommend ddPCR for rapid bedside testing in critically ill ICU patients (Trouchet et al., 2025; Wu et al., 2022). For non-critically ill patients requiring comprehensive pathogen screening without prior etiological clues, or for the targeted detection of specific antimicrobial-resistant pathogens, tNGS may be a suitable option (Wang and Cao, 2024). In contrast, mNGS is particularly valuable for critically ill patients, complex infections, or cases in which conventional diagnostic methods fail to identify rare or unexpected pathogens (Wang and Wang, 2021). Beyond nucleic acid-based detection, emerging metabolic imaging approaches—such as PET tracers that exploit bacterial siderophore systems for copper uptake—represent a complementary strategy for localizing infection foci *in vivo*, though they do not provide pathogen identification at the species level (Siddiqui et al., 2021).

Plasma mNGS has been widely employed for pathogen detection among hematologic disorders. However, studies on the scope of clinical applications of whole blood mNGS remain relatively limited due to its greater technical complexity and higher associated costs and high associated costs. In contrast to previously published literature using plasma mNGS alone (Zou et al., 2024; Huang et al., 2023; Chen et al., 2024), our study demonstrated that whole blood mNGS assays exhibited superior detection performance in patients with non-malignant tumors and those who developed infections following HSCT (Table 2). Furthermore, for BSI and pulmonary infection patients, whole blood mNGS also showed improved diagnostic performance compared with plasma alone and CMT (Figure 5). To our knowledge, this is the first study to specifically evaluate the diagnostic yield of whole blood mNGS for pulmonary infections in patients with hematological diseases. While previous studies have demonstrated the utility of whole blood mNGS in sepsis and bloodstream infections (Wu et al., 2023; Zhu et al., 2025; Chai et al., 2026), its performance in pulmonary infections has not been previously assessed. Our findings therefore provide novel evidence to support the broader clinical application of whole blood mNGS beyond bloodstream-related infections.

Patients with hematologic disorders and suspected infections frequently receive empirical antimicrobial treatment before the causative pathogen is identified. Therefore, we further evaluated the real-world clinical impact of whole blood mNGS on treatment decision-making. In our study, whole blood mNGS positively influenced treatment strategies in 72.73% of patients with suspected infections (Table 3), which is substantially higher than the impact reported for plasma mNGS in previous studies, where positive effects ranged from 7.3% to 38.6% in infected patients (Xu et al., 2023; Hogan et al., 2021). Additionally, compared to plasma mNGS, whole blood mNGS led to beneficial adjustments in clinical medication for an additional 10 patients (4.3% of the cohort), representing a notable improvement over the 18.61% impact observed with BC. Nevertheless, considering its higher cost, whole blood mNGS may be most appropriately reserved for specific clinical scenarios, such as suspected intracellular bacterial infections, or cases in which conventional diagnostic methods (including plasma mNGS) yield negative results despite persistent suspicion of infection.

Previous studies have established that plasma mNGS exhibits robust performance in fungal detection compared to CMT (Zhang et al., 2024; Wang et al., 2022). Fungal infections are a common and serious complication in immunocompromised patients with hematologic disorders, often progressing rapidly if not diagnosed and treated promptly (Shen et al., 2023). Delayed diagnosis and treatment can substantially increase mortality in these patients. In our study, two patients initially tested negative for fungal infections using CMT, but whole blood mNGS identified additional sequences of *R. pusillus* and *A. ruber* in blood cells. Based on these mNGS findings and corresponding radiological evidence, targeted antifungal therapy was initiated. The patients subsequently showed clinical improvement, and fungal infection was later confirmed. These cases underscore the potential diagnostic advantage of whole-blood mNGS for patients with hematologic diseases who are at risk for invasive fungal infections such as mucormycosis or aspergillosis. Specifically, whole-blood mNGS may offer additional diagnostic value over plasma mNGS alone for pathogen identification and antifungal treatment guidance, particularly when empirical or culture-directed therapy is ineffective.

We acknowledge that this study has several limitations and requires further optimization. First, as a single-center retrospective study, although the sample size was relatively substantial, the findings may be subject to selection bias. Additionally, the significant disparity in subgroup sizes could influence the results; thus, future multi-center studies are necessary to validate our conclusions. Therefore, caution should be exercised when extrapolating these findings to patients without hematologic disorders. Second, all patients received early empiric antibiotic therapy prior to whole blood mNGS testing, which might have reduced the sensitivity of both mNGS and CMT. As a result, prospective studies are needed to confirm these findings. Finally, whole blood mNGS is expensive, and although it has better detection performance, its application needs to be considered comprehensively.

In summary, compared with plasma mNGS alone, whole blood mNGS sequencing has a greater potential for detecting pathogens in patients with suspected infections related to hematologic disorders, especially those with non-hematologic malignancies or those undergoing hematopoietic stem cell transplantation. Moreover, whole blood mNGS significantly increased the pathogen detection rate and had a positive impact on the treatment of BSI and pulmonary infection, especially in patients with fungal infection.

## Data Availability

The data presented in the study are deposited in the SRA repository, accession number PRJNA1240079.
