# Supplementary material for: Metagenomics next-generation sequencing of plasma combined with blood cells for improving the prognosis of early infection in patients with hematologic disorders: a real-world cohort study in northern China
Source: Front Mol Biosci. 2026 Jun 10;13:1662559. doi: 10.3389/fmolb.2026.1662559 (PMC13290573; doi:10.3389/fmolb.2026.1662559)
Supplement: Supplementary file 1 [file DataSheet1.docx]

Supplementary Material

## Supplementary Figures


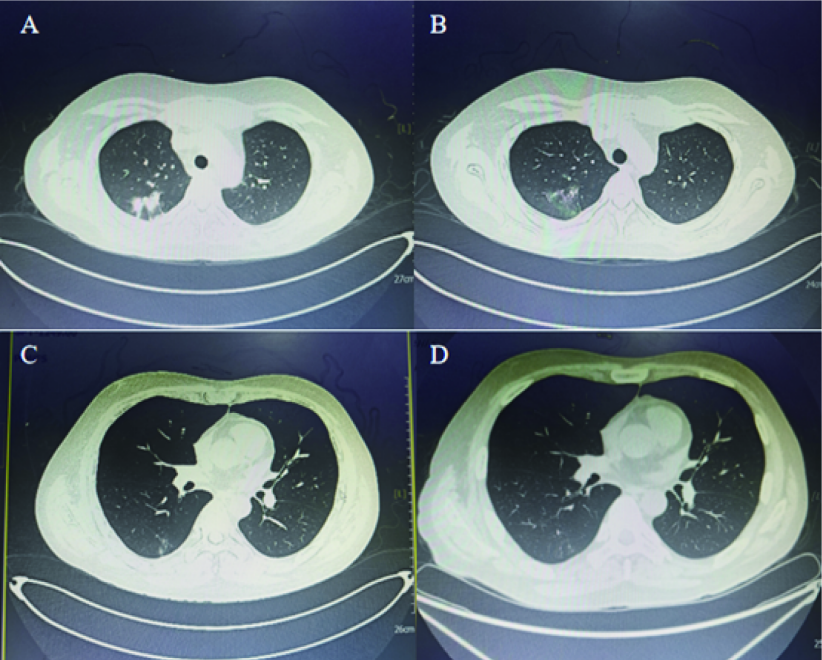


## Supplementary Figure 1. Imaging features for the two fungal infection patients. (A) Patient 1 before antifungal therapy. (B) After antifungal therapy in Patient 1. (C) Patient 2 before antifungal therapy. (D) After antifungal therapy in Patient 2.

## Supplementary Tables

Supplementary table 1. Pathogen spectrum detected by different methods among infected hematologic disorder patients.

| **Pathogen** | **Category** | **Plasma mNGS only** | **Whole blood mNGS** | **BC** | **CMT** | **Total** |
| --- | --- | --- | --- | --- | --- | --- |
| *Pseudomonas aeruginosa* | Bacteria | **16** | **26** | 2 | **15** | **59** |
| *Klebsiella pneumoniae* | Bacteria | 12 | **23** | **14** | **17** | **66** |
| *Enterococcus faecium* | Bacteria | **13** | 19 | 4 | 8 | 44 |
| *Escherichia coli* | Bacteria | 9 | 14 | **6** | 6 | 35 |
| *Enterococcus faecalis* | Bacteria | 7 | 10 | 1 | 1 | 19 |
| *Stenotrophomonas maltophilia* | Bacteria | 6 | 8 | 2 | **9** | 25 |
| *Staphylococcus aureus* | Bacteria | 2 | 8 | 3 | 3 | 16 |
| *Enterobacter hormaechei* | Bacteria | 4 | 6 | 1 | 1 | 12 |
| *Acinetobacter baumannii* | Bacteria | 3 | 4 | 1 | 3 | 11 |
| *Staphylococcus hominis* | Bacteria | 1 | 4 | 1 | 1 | 7 |
| *Bacteroides fragilis* | Bacteria | 4 | 4 | 1 | 1 | 10 |
| *Staphylococcus capitis* | Bacteria | 1 | 3 |  |  | 4 |
| *Proteus mirabilis* | Bacteria | 2 | 3 |  | 2 | 7 |
| *Staphylococcus haemolyticus* | Bacteria | 2 | 3 | 1 | 2 | 8 |
| *Leuconostoc lastis* | Bacteria | 2 | 2 |  |  | 4 |
| *Staphylococcus epidermidis* | Bacteria | 1 | 2 | 1 | 1 | 5 |
| *Streptococcus pneumoniae* | Bacteria | 2 | 2 |  |  | 4 |
| *Streptococcus oralis* | Bacteria | 2 | 2 |  |  | 4 |
| *Klebsiella michiganensis* | Bacteria |  | 2 |  |  | 2 |
| *Burkholderia cenocepacia* | Bacteria | 1 | 2 |  |  | 3 |
| *Acinetobacter pittii* | Bacteria | 1 | 2 |  | 1 | 4 |
| *Other* | Bacteria | 29 | 42 | 6 | 6 | 83 |
|  |  |  |  |  |  | 0 |
| *Mycobacterium tuberculosis complex* | Special pathogen | 2 | 4 |  |  | 6 |
| *Ureaplasma Urealyticum* | Special pathogen | 1 | 1 |  |  | 2 |
| *Mycoplasma hyorhinis* | Special pathogen | 1 | 1 |  |  | 2 |
|  |  |  |  |  |  | 0 |
| *Aspergillus fumigatus* | Fungi | **6** | 8 |  |  | 14 |
| *Pneumocystis jirovecii* | Fungi | 2 | 4 |  |  | 6 |
| *Lichtheimia ramose* | Fungi | **3** | 3 |  |  | 6 |
| *Rhizomucor pusillus* | Fungi | 2 | 2 |  |  | 4 |
| *Trichosporon asahii* | Fungi | 1 | 2 |  |  | 3 |
| *Rhizopus microsporus* | Fungi | 2 | 2 |  |  | 4 |
| *Candida parapsilosis* | Fungi |  | 2 |  |  | 2 |
| *Aspergillus chevalieri* | Fungi | 1 | 2 |  |  | 3 |
| *Fusarium verticillioides* | Fungi | 1 | 2 |  |  | 3 |
| *Aspergillus flavus* | Fungi | 2 | 2 | 0 | 1 | 5 |
| *Other* | Fungi | 8 | 12 | 6 | 6 | 32 |
|  |  |  |  |  |  | 0 |
| *Human betaherpesvirus 5* | Virus | **12** | **32** |  | 9 | **53** |
| *Human alphaherpesvirus 1* | Virus | **9** | 11 |  |  | 20 |
| *Human polyomavirus 1* | Virus | 4 | 8 |  |  | 12 |
| *Human gammaherpesvirus 4* | Virus | 3 | 7 |  | 1 | 11 |
| *Human betaherpesvirus 6B* | Virus | 3 | 5 |  |  | 8 |
| *Human adenovirus C* | Virus |  | 3 |  |  | 3 |
| *Human parvovirus B19* | Virus |  | 2 |  |  | 2 |
| *Hepatitis B virus* | Virus |  | 2 |  |  | 2 |
| *Other* | Virus | 3 | 3 |  | 2 | 8 |
|  |  |  |  |  |  | 0 |
| *Leishmania donovani* | Parasite | 1 | 1 |  |  | 2 |
| *Leishmania infantum* | Parasite |  | 1 |  |  | 1 |

Note: The number of detection for the most common pathogens is marked in bold red font.
